# Identification and Characterization of DNA Demethylase Genes and Their Association With Thermal Stress in Wheat (*Triticum aestivum* L.)

**DOI:** 10.3389/fgene.2022.894020

**Published:** 2022-07-22

**Authors:** Vijay Gahlaut, Harsha Samtani, Tinku Gautam, Paramjit Khurana

**Affiliations:** ^1^ Department of Plant Molecular Biology, University of Delhi, New Delhi, India; ^2^ CSIR-Institute of Himalayan Bioresource Technology, Palampur, India; ^3^ Department of Genetics and Plant Breeding, Chaudhary Charan Singh University, Meerut, India

**Keywords:** DNA demethylation, bread wheat, heat stress response, cis-regulatory elements, simple sequence repeats (SSR)

## Abstract

DNA demethylases (dMTases) are essential proteins in plants that regulate DNA methylation levels. The dMTase genes have been explored in a number of plant species, however, members of this family have not been reported in wheat. We identified 12 wheat dMTase genes divided into two subfamilies: repressor of silencing 1 (ROS1) and DEMETER-Like (DML). The TadMTases in the same subfamily or clade in the phylogenetic tree have similar gene structures, protein motifs, and domains. The promoter sequence contains multiple *cis*-regulatory elements (CREs) that respond to abiotic stress, hormones, and light, suggesting that the majority of *TadMTase* genes play a role in wheat growth, development, and stress response. The nuclear localization signals (NLSs), subcellular localization, and SRR motifs were also analyzed. The expression profile analyses revealed that *TadMTase* genes showed differential gene expression patterns in distinct developmental stages and tissues as well as under heat stress (HS). Furthermore, the qRT-PCR analysis revealed that *TadMTase* gene expression differed amongst wheat cultivars with varying degrees of HS tolerance. Overall, this work contributes to the understanding of the biological function of wheat dMTases and lays the foundation for future investigations.

## Introduction

DNA methylation, along with other epigenetic components (histone modifications, chromatin remodeling, and non-coding small RNAs), regulates the transcription dynamics of several downstream genes and affects the development and stress response in plants (Zhang et al., 2018). DNA methylation at cytosine (C) residues in plants is established through the RNA-directed DNA methylation (RdDM) pathway in three sequence contexts: CG, CHG, and CHH (H stands for A/C/T) (Chan et al., 2005). Four different kinds of methyltransferases have established and maintained C methylation, that is, methyltransferase (MET), chromomethylase (CMT), domain rearranged methyltransferase (DRM), and DNA methyltransferase homologue 2 (DNMT2) (Bender, 2004; Chan et al., 2005; Law and Jacobsen, 2010; Gahlaut et al., 2020).

The homeostasis of DNA methylation also needs the active involvement of DNA demethylase (dMTase) enzymes, which remove C methylation *via* the base excision repair (BER) pathway (Gong et al., 2002; Zhu, 2009; Mok et al., 2010). In Arabidopsis, four dMTase enzymes (DNA glycosylase/lyases), that is, DEMETER (DME), REPRESSOR OF SILENCING 1 (ROS1), DEMETER-LIKE (DML2) and DML3 have been reported (Choi et al., 2002; Gehring et al., 2006; Penterman et al., 2007; Ortega-Galisteo et al., 2008; Zhu, 2009; Zhang et al., 2018). The ROS1 and DML dMTases are expressed in all vegetative tissues, including root and shoot tissues (Penterman et al., 2007; Calarco et al., 2012), whereas DME is mainly expressed in companion cells of the gametes tissues (Penterman et al., 2007; Huh et al., 2008). In Arabidopsis, it was shown that DME is required for sporophyte development and plays an essential role in the maintenance of stem cell activities during the sporophytic life cycle (Kim et al., 2021). Furthermore, it was demonstrated that dMTases (ROS1 and DME) were also involved in regulating seed development in rice and maize (La et al., 2011; Ono et al., 2012; Kapazoglou et al., 2013). The null mutation of *ROS1a* dMTase in rice causes abnormal early-stage endosperm development and affects the pollen gametophytic transmission (Ono et al., 2012; Kim et al., 2019). Another dMTase encoding gene, *DNG701* in rice, demethylates the *Tos17* and plays a critical role in seed development (La et al., 2011). The active DNA demethylation executed by *OsROS1* also regulates the number of aleurone cell layers in rice (Liu et al., 2018b). Three rice dMTase genes (*DNG702, DNG701,* and *DNG704*) have recently been shown to demethylate DNA at different genomic locations in gametes and zygotes. These findings suggest that active DNA demethylation is crucial for zygotic gene expression and reproduction (Zhou et al., 2021). Recently, it was shown that active DNA demethylation of *RESISTANCE METHYLATED GENE 1* (*RMG1*) and *RECEPTOR-LIKE PROTEINS 43* (*RLP43*) promoters by ROS1 regulates transcriptional immune reprogramming and provides disease resistance in plants (Halter et al., 2021). In barley, a DME glycosylase (*HvDME*) was characterized and the transcriptomic studies during seed development and under dehydration stress revealed that *HvDME* is involved in seed maturation and drought stress response (Kapazoglou et al., 2013). Plants’ abiotic stress response regulation also requires active DNA demethylation. For instance, in rice, the salt-sensitive variety IR29 showed induced expression of dMTase genes (*DNG701* and *DNG710*) in response to salinity stress (Ferreira et al., 2015). Similarly, in tomatoes, it was observed that expression of the *DML2* gene is downregulated upon cold stress treatment (Zhang et al., 2016). DNA demethylation was also shown to have a role in the regulation of plant heat stress (HS) responses. For example, in Arabidopsis, it was shown that HS causes a reduction in DNA methylation levels in stress responsive genes (*HSP70*, *RPL26A*, and *POX1*). This demonstrated that the active demethylation process was triggered by HS (Korotko et al., 2021). DNA demethylation has also been linked to seed germination regulation during HS (Malabarba et al., 2021). The above results indicate that active DNA demethylation maintained by dMTases is a crucial process involved in various biological processes, including thermal stress regulation in plants. Apart from this, the role of DNA methylation has also been implicated in the stress-induced formation of the quality-related metabolites in tea (Yang et al., 2021a). Interestingly, the DNA methylation levels of key biosynthesis gene of indole, that is, tryptophan synthase β-subunit 2 were found to be reduced during the continuous wounding stress which occurs during oolong tea processing stage. This promoted the binding of CsMYC2a to the promoter of this gene, which caused the increased accumulation of the aromatic indole compound in the tea (Yang et al., 2021b). Similarly, in the case of Arabidopsis, under heat stress conditions, DNA demethylation has been found to be responsible for seed germination (a process where metabolites accumulated during maturation are used) (Malabarba et al., 2021). Thus, it could be speculated that a similar mechanism might also exist in wheat where DNA methylation affects the quality of wheat seeds by regulating the metabolites.

Wheat (*Triticum aestivum* L.) has been one of the world’s most important agricultural crops, accounting for around 30% of global grain output (FAO, 2021). Recently, it was projected that global surface temperatures will rise by 1.5°C in the next 20 years (by 2040), resulting in a major decrease in worldwide wheat productivity (IPPC, 2021). Therefore, to ensure wheat yield, researchers must analyze genomic regions that regulate HS tolerance in wheat. The characterization of wheat dMTases becomes important as their roles in plant development and stress responses are still largely unknown.

In the present study, using the most recent wheat genome sequences, we systematically identified 12 dMTase genes. The chromosome localization, evolutionary relationship, gene structures, conserved domains, motifs, SSR motifs, *cis*-regulatory elements, and subcellular localization were analyzed. Finally, the expression profiles of *TadMTases* were investigated in various plant tissues and development stages, as well as in response to heat stress. Altogether, these findings contribute to our understanding of the structure, phylogeny, and significant regulatory functions of wheat dMTase genes. Furthermore, our findings lay the foundation for possible functional investigation of these genes in order to improve wheat heat stress resistance.

## Materials and Methods

### Sequence Retrieval and Identification of dMTases in Wheat

To obtain wheat sequences of dMTases, we constructed a local protein database, which contains all of the wheat protein sequences accessible on the Ensemble database available at http://plants.ensembl.org/index.html (Howe et al., 2020). Then, the database was examined with known Arabidopsis dMTase proteins (retrieved from the TAIR database, https://www.arabidopsis.org) utilizing the BLAST_P_ program with an e-value of le-5 and an identity of 50%. The dMTase genes were cross-checked and redundant sequences were eradicated. Using the SMART online software program (http://smart.embl-heidelberg.de/) (Schultz et al., 2000) and the Pfam database (http://pfam.xfam.org/) (El-Gebali et al., 2018), all of the detected wheat dMTase protein sequences were also validated for the presence of conserved domains (HhH-GPD glycosylase domain; PF00730 and the RNA-recognition motif in Demeter; PF15628). The ExPASy server (http://www.expasy.org/) (Artimo et al., 2012) was used to determine the relative molecular weight (MW) and isoelectric point (PI) of TadMTase proteins.

### Chromosomal Distribution, Gene Structure, and Motif Analysis

The *TadMTase* genes were assigned to individual chromosomes using information available in the Ensemble database (http://plants.ensembl.org) (Howe et al., 2020). The chromosomal location of TadMTase genes was drawn using TBtools software (Chen et al., 2020). The gene structures were also analyzed using the TBtools software. The motifs of TadMTase were analyzed using the MEME suite (Bailey et al., 2009), and their positions were displayed using TBtools software.

### Multiple Sequence Alignment and Phylogenetic Tree Construction

The dMTase protein sequences of wheat (12), *Aegilops tauschii* (4), *Triticum urartu* (3), *Triticum turgidum* (7), *Oryza sativa* (4), *Arabidopsis thaliana* (4), *Gossypium raimondii* (4), *Gossypium arboretum* (5), *Gossypium barbadense* (6), and *Gossypium hirsutum* (10) were aligned using the MUSCLE package available at MEGA X software (Stecher et al., 2020). The phylogenetic tree was created using the neighbor-joining method with the Poisson model, pair-wise deletion, and 1,000 bootstrap values in MEGA X software. The phylogenetic tree was visualized using the iTOL online tool (Letunic and Bork, 2021). Dated phylogeny trees for 31 plant species were retrieved from TimeTree (Kumar et al., 2017).

### Analysis of *Cis*-Regulatory Elements

To analyze the *cis*-regulatory elements (CREs) of TadMTase, the upstream sequences (1,500 bp) of the start codon were fetched from the Ensemble database (Howe et al., 2020). The CREs were identified using the PlantCARE online server (Lescot et al., 2002).

### Nuclear Localization Signals, Subcellular Localization, and Gene Ontology Analysis

The NLS in TadMTases was predicted using the cNLS Mapper online tool (Kosugi et al., 2009). Using the CELLO online resource (Yu et al., 2014), the subcellular localization of TadMTases was predicted. The corresponding Ensemble database IDs of TadMTase genes were subjected to the ShinyGO v0.61 database (Ge et al., 2020) to obtain gene ontology (GO) annotation.

### Identification of Simple Sequence Repeats and Primer Designing

The WebSat online tool (Martins et al., 2009) was used to mine simple sequence repeats (SSRs) in the TadMTase gene sequences. The minimum length criteria of six for di-nucleotide repeats and three for tri-nucleotide repeats, tetra-nucleotide repeats, penta-nucleotide repeats, and hexa-nucleotide repeats. The Primer 3 software (available on the WebSat online tool) was used to design primers. The SSR primers were developed using the following criteria: melting temperature (Tm) of 55°C–60°C, primer length of 20–25 bp, and product size ranging from 110 to 400 bp.

### Expression Profiling of DNA Demethylase Genes

The expression levels of *TadMTase* genes in different tissues, developmental stages, and responses to heat stress were analyzed using the Genevestigator tool (Hruz et al., 2008). The Genevestigator has a broad collection of public microarrays and RNA-Seq study data.

### Plant Materials and Heat Stress Treatment

Expression profiling of *TadMTase* genes was examined in two wheat cvs. HD2329 (heat-sensitive) and HD2985 (heat-tolerant) under control and heat stress conditions. Seeds of two cultivars were surface-sterilized with 1% hydrogen peroxide, washed gently with distilled water, and germinated for 2 days in Petri plates on water-soaked filter paper at 22°C. Then the seedlings were transferred and cultivated in half-strength Hoagland nutrient solution at 22°C with a 16:8 h light/dark photoperiod. HS was induced by exposing one-week-old seedlings to a temperature of 42°C for 2 h (Meena et al., 2022). The environment for the control sample was set to 22°C. Each treatment (control and HS treated) included three biological replicates. All samples were promptly frozen using liquid nitrogen and preserved at −80°C for RNA extraction.

### RNA Isolation and Quantitative Real-Time PCR

Total RNA was extracted from 100 mg tissues using TRizol reagent (Ambion), and DNA was removed by DNaseI enzyme (TaKaRa, United States). cDNA was generated using the RevertAid First Strand cDNA Synthesis Kit (Thermo Scientific). Quantitative expression was performed with a QuantStudio Real-Time PCR system (Applied Biosystems, United States). Three technical replicates for each sample were used. The details of gene-specific primers are provided in Supplementary Table S1. The wheat *GAPDH* gene was used as an internal reference gene and the relative gene expression was estimated using the 2^−ΔΔCT^ method (Livak and Schmittgen, 2001).

## Results

### Identification of dMTase Genes in Wheat

After examining the wheat reference genome, a total of 12 full-length dMTase genes were identified. The nomenclature of 12 wheat dMTase genes was based on the corresponding genes reported for rice and Arabidopsis. The 12 *TadMTase* genes were mainly divided into two groups (ROS1 and DML). The *TaROS1a* (5A/5B/5D) genes were homologous to *AtROS1* (AT2G36490) and *OsROS1a* (Os01t0218032) in Arabidopsis and rice respectively; the *TaROS1c1* (1A/1B/1D) and *TaROS1c2* (1A/1B/1D) genes were homologous to *AtROS1* (AT2G36490) and *OsROS1c* (Os05t0445900) in Arabidopsis and rice respectively; the *TaDML3a* (3A/3B/3D) genes were homologous to *AtDML3* (AT4G34060) and *OsDML3a* (Os02t0496500) in Arabidopsis and rice respectively (see Supplementary Table S2). The length of the transcript of *TadMTase* genes ranged from 3,003 bp (*TaDML3a-3D*) to 7,285 bp (*TaROS1a-5D*), and the average length was found to be 5,377 bp. The protein length ranged from 989 aa (*TaDML3a-3A*) to 1982 aa (*TaROS1a-5B*), and the average length was 1,581 aa. The estimated molecular weights of each TadMTase protein varied from 109.6 to 218.4 kDa (Table 1). The dMTase gene names, gene IDs, chromosomal location and other features are summarized in Table 1.

**TABLE 1:** Summary and characteristics of dMTase genes identified in wheat.

Gene name	Gene ID^a^	Chromosome location	Transcript length (bp)	No. of exons	Protein			Subcellular localization
					Length (aa)	pI	Mw (kDa)	
*TaROS1a-5A*	TraesCS5A02G169000	5A:360071286-360084523	6,607	18	1,975	6.0	217.3	Nuclear
*TaROS1a-5B*	TraesCS5B02G165800	5B:307397203-307412033	7,176	18	1,982	6.1	218.4	Nuclear
*TaROS1a-5D*	TraesCS5D02G173300	5D:270261993-270276412	7,285	18	1,981	6.1	218.0	Nuclear
*TaROS1c1-1A*	TraesCS1A02G349600	1A: 534535188-534547681	5,232	20	1,595	6.8	177.8	Nuclear
*TaROS1c1-1B*	TraesCS1B02G364100	1B: 593446994-593459819	5,525	19	1,600	6.5	178.0	Nuclear
*TaROS1c1-1D*	TraesCS1D02G352500	1D: 437848604-437860415	5,770	19	1,586	6.8	176.1	Nuclear
*TaROS1c2-1A*	TraesCS1A02G278000	1A:473630543-473641488	5,775	21	1,765	7.4	195.4	Nuclear
*TaROS1c2-1B*	TraesCS1B02G286900	1B:498862239-498872297	5,740	22	1,769	7.0	196.3	Nuclear
*TaROS1c2-1D*	TraesCS1D02G277100	1D:374039261-374049890	5,497	19	1,734	6.7	192.3	Nuclear
*TaDML3a-3A*	TraesCS3A02G022500	3A:12776134-12784267	3,429	17	989	6.9	109.6	Nuclear
*TaDML3a-3B*	TraesCS3B02G023200	3B:9931500-9939414	3,490	17	1,000	6.8	110.7	Nuclear
*TaDML3a-3D*	TraesCS3D02G024100	3D:7605843-7613892	3,003	17	1,000	6.9	110.6	Nuclear

a ID available at Ensemble plant database (https://plants.ensembl.org/index.html).

### Chromosome Locations of Wheat dMTases

The 12 wheat dMTases were located on nine (1A, 1B, 1D, 3A, 3B, 3D, 5A, 5B, and 5D) of the 21 wheat chromosomes (Supplementary Figure S1). Furthermore, a maximum of six genes were located on group 1 chromosomes (1A/1B/1D), whereas a minimum of three genes were mapped onto group 5 and 3 chromosomes. Since bread wheat is a hexaploid species (i.e., it contains three sub-genomes). Three homologues for each gene were found corresponding to chromosomes A, B, and D (Table 1; Supplementary Figure S1).

### Phylogenetic Relationship of dMTases

**FIGURE 1:**
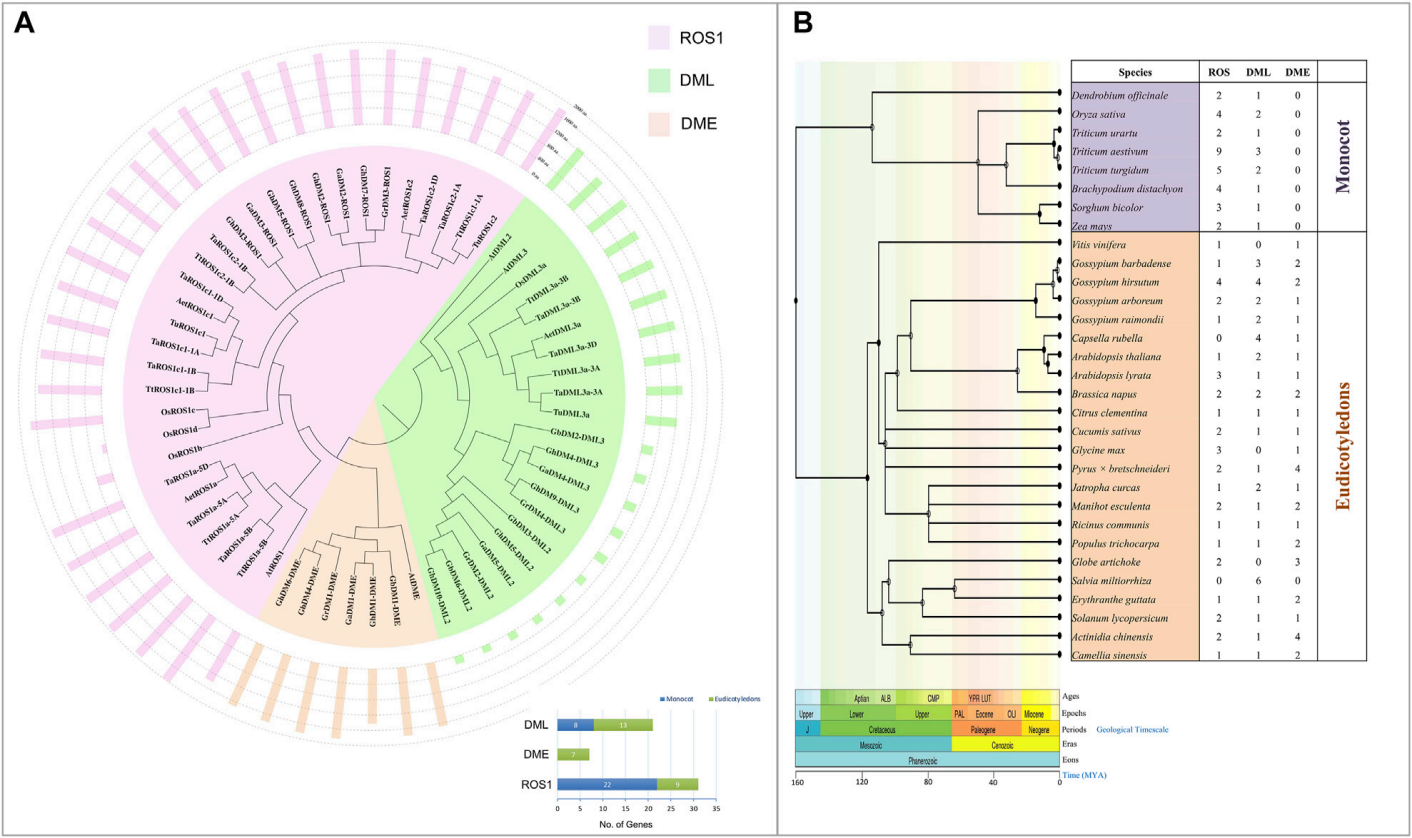
Evolutionary relationship among various dMTases. **(A)** Evolution relationship among the 10 plant species (*Ta*, *Triticum aestivum*; *Aet*, *Aegilops tauschii*; *Tu*, *Triticum Urartu*; *Tt*, *Triticum turgidum*; *At*, *Arabidopsis thaliana*; *Os*, *Oryza sativa*; *Ga*, *Gossypium arboretum*; *Gb*, *Gossypium barbadense*; *Gh*, *Gossypium hirsutum*; *Gr*, *Gossypium raimondii*). Graph (present at the lower right corner) shows the number of dMTases in each sub-family (ROS1, DML, and DME). **(B)** Dated phylogeny trees for 31 plant species belonging to monocot and eudicotyledons. The number of genes present/absent of ROS1, DML, and DME in different plant species is shown in the columns. MYA, a million years ago.

To determine the phylogenetic relatedness of dMTases in plants, 59 dMTase protein sequences from five monocots (*Triticum aestivum*, *Aegilops tauschii, Triticum urartu, Triticum turgidum, and O. sativa*) and five dicotyledons (*Arabidopsis thaliana*, *Gossypium hirsutum*, *Gossypium arboretum*, *Gossypium raimondii, and Gossypium barbadense*) were used for multiple sequence alignments and to build a phylogenetic tree (Figure 1A). All the members of dMTase are grouped into three subfamilies/clades: ROS1, DME, and DML. The ROS1 clade comprised 22 monocots and nine dicots, the DML clade comprised eight members of monocots and 13 dicots, while the DME comprised seven members of dicots only (Figure 1A). Furthermore, we also constructed an evolutionary tree of life for 31 plant species belonging to monocot (8) and dicot (23) and showed the total number of dMTase proteins for each species (Figure 1B). Our analysis revealed that the DME subfamily of dMTases was absent in monocots and existed only in dicots.

### Gene Structure, Motif and Domain Composition of TadMTases

**FIGURE 2:**
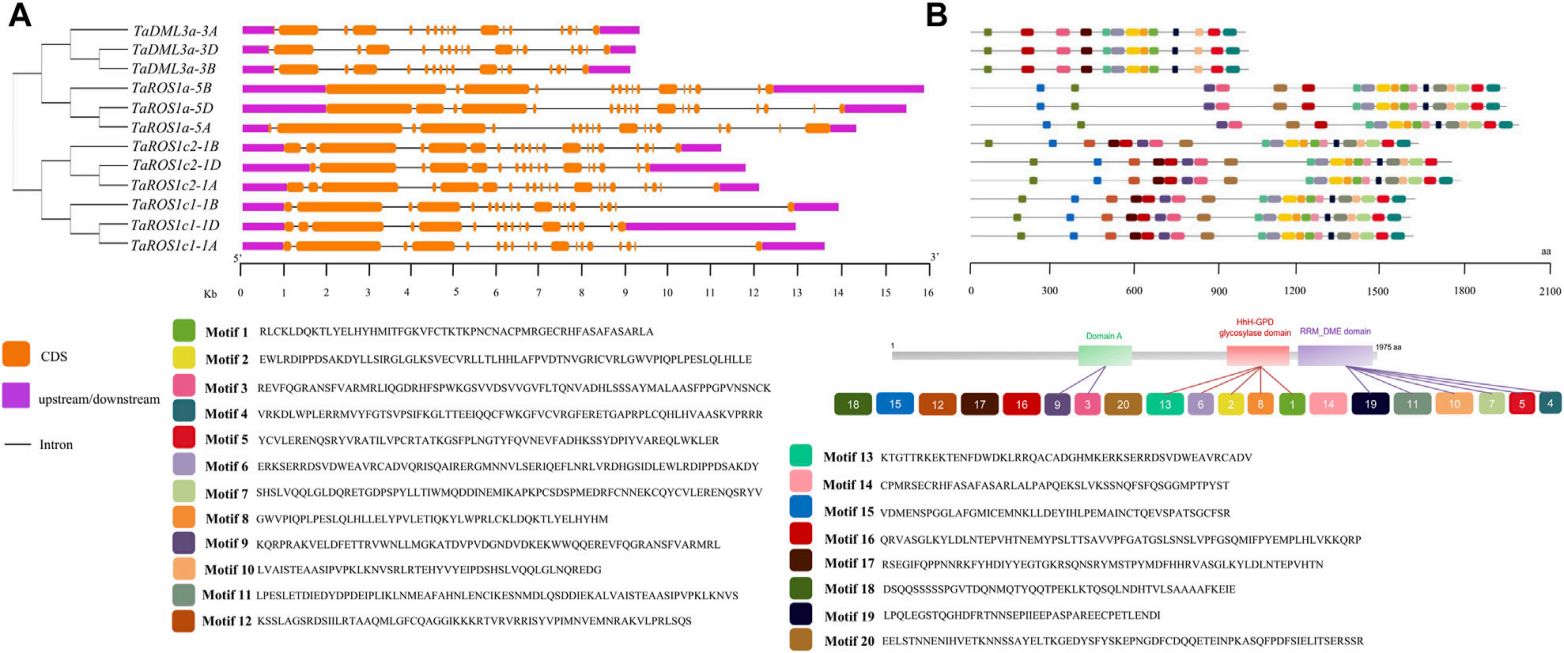
Exon-intron structures and conserved motif compositions of TadMTases. **(A)** Exon–intron structures of *TadMTase* genes. Exons are shown as orange boxes, introns are denoted by thin dark grey lines, and upstream/downstream regions are shown as purple boxes. The lengths of exons and introns can be determined using the scale bar on the bottom. **(B)** Motifs organization of TaDMTase proteins. Color boxes represent the position of different motifs, and box sizes show the length of motifs. Sequences for 20 different motifs were also provided on the right side.

To gain further insights into the *TadMTase* genes, we surveyed the gene structure, conserved domain, and motif components of each *TadMTase* gene. The number of exons in the *TadMTase* genes ranged from 17 to 22 (Figure 2A; Table 1). We found that the number of exons in *dMTase* genes from the same homoeologous group did not differ much (maximum up to three exons in *TaROS1c2*). Among these, the dMTase gene with the maximum number (22) of exons was found to be *TaROSc2*-*1B*, while *TaDML3a* had the least number of exons (17).

The distribution of conserved motifs in the TadMTase proteins was predicted using the MEME online suite. Twenty different types of motifs, which were designated 1 to 20, were predicted in each TadMTase protein. The amino acid length of motifs varied from 21 to 50. The number of motifs in each dMTase varied from 13 to 20. Among them, motifs 3 and 9 were part of Domain A, motifs 1, 2, 6, 8, and 13 were part of the HhH-GPD glycosylase domain, and motifs 4, 5, 7, 10, 11, and 19 were part of the RRM_DME domain (Figure 2B). Furthermore, the Motifs 1, 2, 3, 4, 5, 10, 11, 13, 16, 18, and 19 were highly conserved and found in every dMTase protein. However, some motifs (9, 15, and 20) were specific to the ROS gene group (Figure 2B). It could be inferred that motifs found in all dMTase proteins are possibly associated with conserved functions, but those specific to the ROS gene group may be involved in gene-specific functions.

**FIGURE 3:**
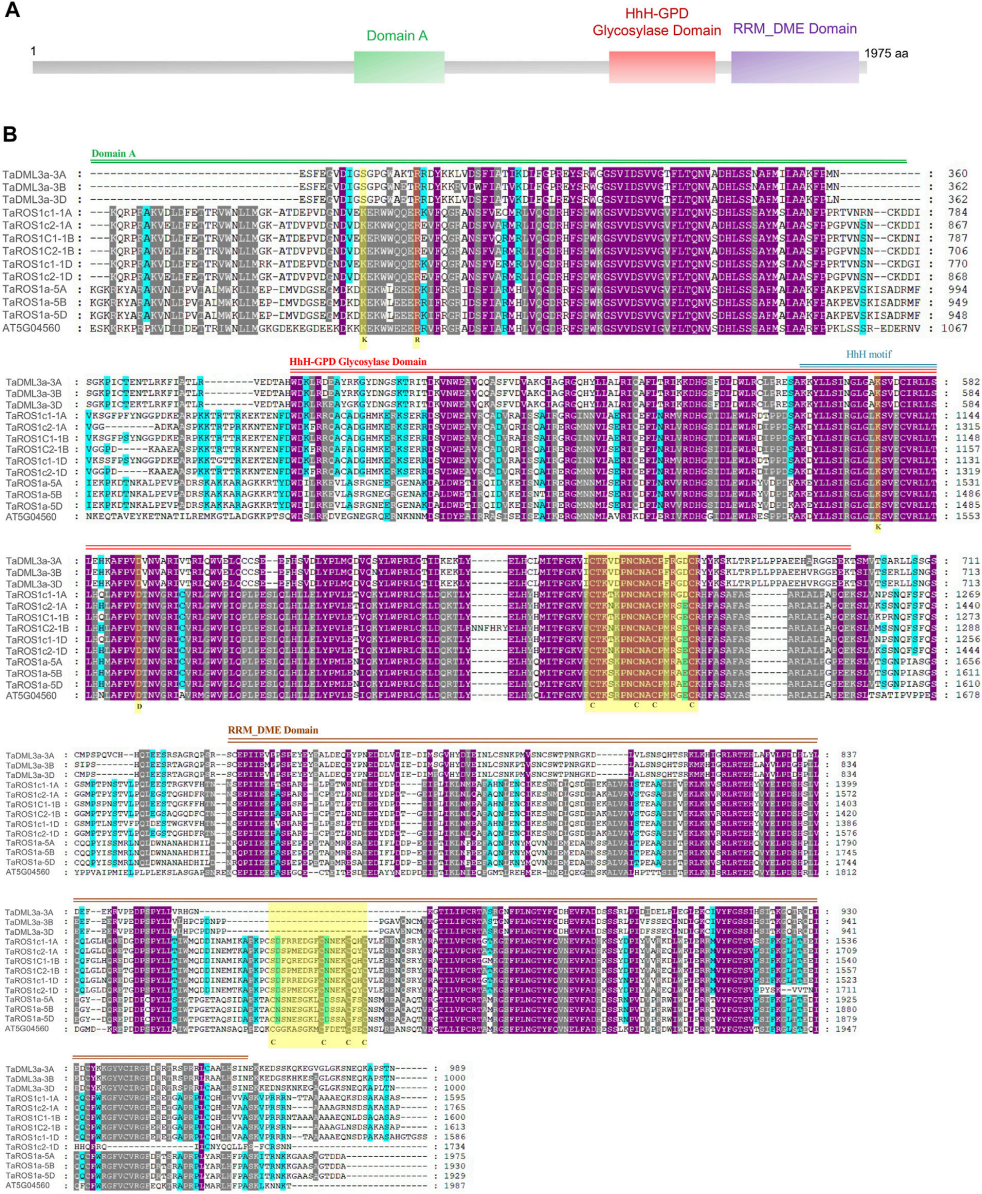
Conserved domain composition and multiple sequence alignments of the conserved domain sequences of *TaDMTase* proteins. **(A)** Three different conserved domains (Domain A, HhH-GPD glycosylase domain and RRM_DME domain) are shown in different colored boxes. **(B)** Multiple sequence alignment analysis of the conserved domain sequences of 12 wheat dMTase protein sequences and an Arabidopsis dMTase protein. The key conserved domains are highlighted by double lines (Domain A by green color, HhH-GPD glycosylase domain by red color and RRM_DME domain by brown color) and the domain names are shown at the top of the sequence. Gene names are shown on the left.

To further determine the structural characteristics of the wheat dMTase family members, conserved domains were also identified. All the wheat dMTases contained three different domains, including 1) Domain A, 2) HhH-GPD glycosylase domain (PF00730) and 3) Domain B or RRM_DME (RNA-recognition motif in Demeter; PF15628) domain (Figure 3). The domain A contains the stretch of basic amino acids (K and R) that is required for DNA binding activity. The HhH-GPD glycosylase domain contains a helix–hairpin–helix (HhH) motif, a glycine/proline-rich loop with a conserved aspartic acid (GPD), and four cysteine residues in the Fe-S cluster, these are involved in 5-methylcytosine excision activity. The RRM_DME domain facilitates the interaction of the catalytic domain with ssDNA or regulatory RNA. The multiple sequence alignments of wheat dMTases showed that these three domains are conserved (Supplementary Figure S2).

### SSR Motifs and Markers’ Development

Out of 12 wheat *dMTase* gene sequences screened for SSRs, ten genes have 17 SSR motifs. The tri-nucleotide repeats motif were found most abundant, comprising about nine (53%) followed by the tetra-nucleotide repeat motifs comprising four (23.5%), whereas, di- and penta-nucleotides SSR formed a small share having 2 (11%) each (Supplementary Table S3). The number of repetitions of a motif ranged from 3 to 19. For each of the 17 SSR motifs, primer pairs were also designed. The following details, such as nucleotide sequence, melting temperature, and product size related to SSR motif primers, are provided in Supplementary Table S3.

### NLS Prediction and Sub-Cellular Localization

All TadMTase members contain both monopartite and bipartite NLSs (except *TaROS1a*-*5A*). In total, 12 dMTases were predicted with 35 NLS (21 mono and 14 bipartite NLSs) (Supplementary Table S4). Subcellular localization prediction indicated that all the 12 wheat dMTase proteins are localized in the nucleus (Table 1).

### *Cis*-Regulatory Elements in the Promoter of TadMTases

**FIGURE 4:**
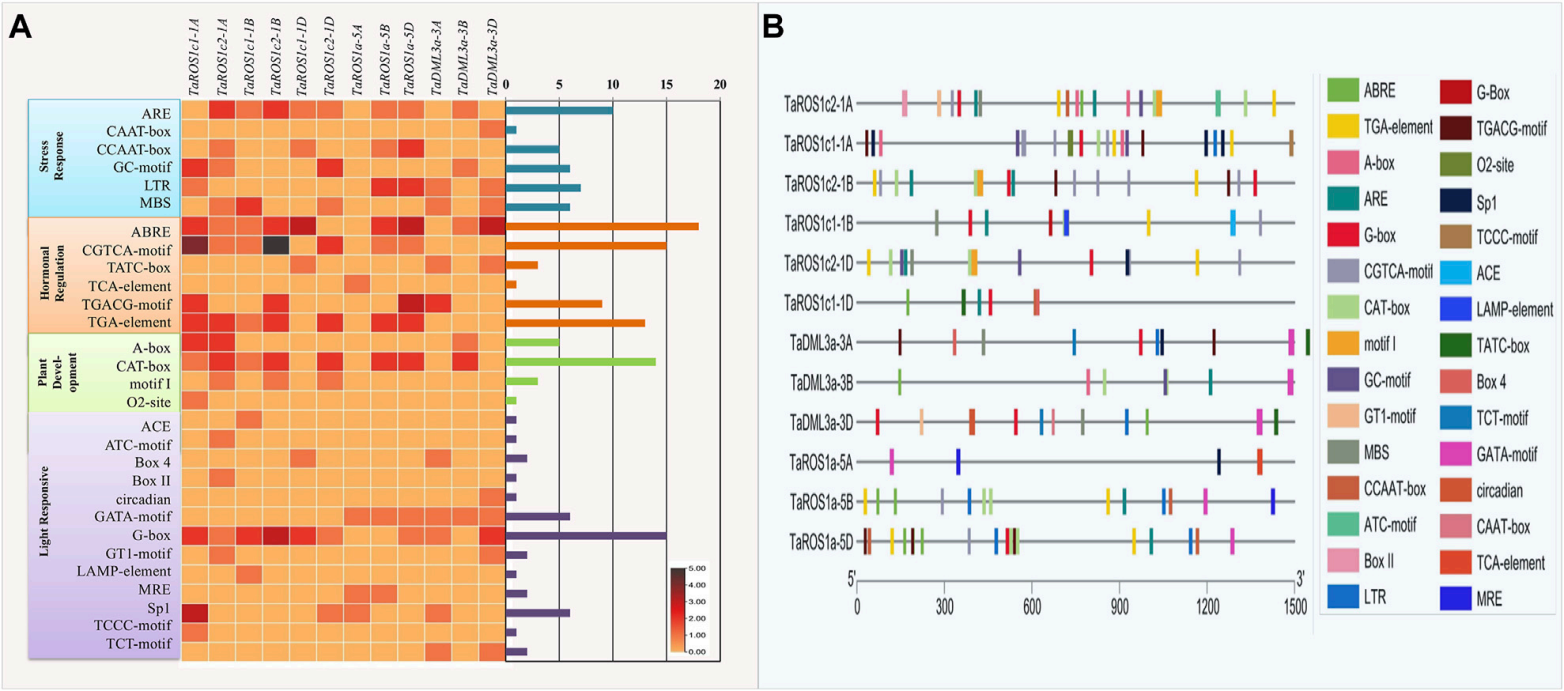
Cis-regulatory elements (CREs) of wheat dMTases genes. **(A)** Showing the number of CREs belonging to the following four categories (stress-responsive, hormonal regulation, plant development, and light-responsive) per dMTase gene as a heatmap. The bar plots on the right represent the number of CREs for each dMTase gene. **(B)** The distribution of different CREs in the promoter region (1,500 base pairs) of wheat dMTase genes. Different CREs are represented by distinct colors as indicated.

To further understand the possible regulatory function of dMTases in plant growth and stress response, we searched for CREs in the 1,500 bp promoter region of wheat *dMTase* genes. A total of 158, representing 29 types of CREs, were predicted (Figure 4). Most of the identified CREs were hormone response factors (37.34%), followed by light-responsive elements (25.94%), stress response elements (22.15%), and plant development-related elements (14.55%). The CREs involved in hormonal signaling comprised auxin-responsive (TGA-element), abscisic acid-responsive (ABRE), methyl jasmonate responsive (CGTCA and TGACG motif), gibberellins responsive (TATC-motif) and salicylic acid-responsive (TCA-element) elements. Among these CREs, the CGTCA, ABRE, TGACG, and TGA motifs appeared to be the most prevalent and were detected in all TadMTase genes. In TadMTase promoters, all 13 kinds of light-responsive CREs were also found. Out of those, G-box CREs appeared to be the most prominent. Each TadMTase gene had one to three G-box copies (Figure 4A).

Thirty-five stress-responsive CREs were found in *TadMTase* genes and out of those, ARE (anaerobic responsive elements) elements comprised 28% of them (10). AREs were most commonly detected at the TadMTase gene promoters. The CAAT-box, CCAAT-box, GC-motif, LTR (low temperature responsive) and MBS were among the other stress-related CREs detected. The GC-motif and CAAT-box have been linked to a variety of abiotic stressors. MYB binding sites (MBS) are known to control drought stress, ARE are known to regulate anaerobic induction under oxidative stress, and LTRs regulate the temperature response.

Furthermore, 24 CREs were discovered that were involved in the growth and development of plants. These included A-Box (5), CAT-box (14), motif I (3), and O2 site (1) (Figure 4A). The CAT-box element appeared to be the most frequent and was detected in ROS and DML dMTase genes. We also found Motif-I, a root-specific regulatory element only in the ROS sub-group genes. Overall, these findings reveal that TadMTase genes may be involved in a variety of abiotic stress responses as well as plant growth regulation.

### Gene Ontology Annotation of TadMTases

**FIGURE 5:**
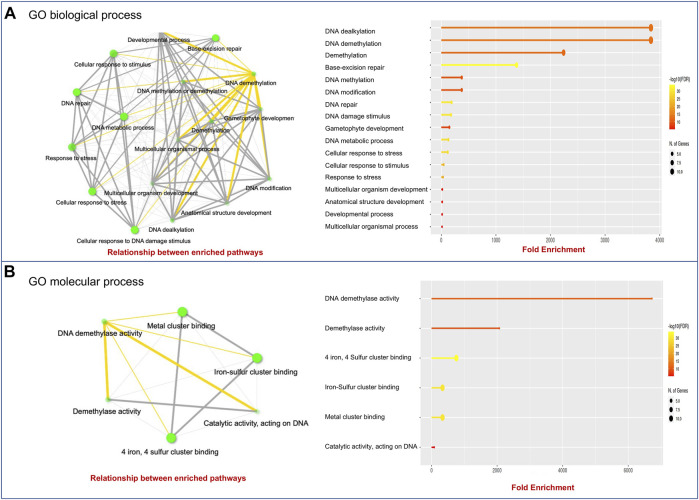
GO analysis using ShinyGo identified enriched biological processes **(A)** and molecular processes **(B)** of dMTase genes. The green dots represent the nodes for each GO biological process or molecular process, while the lines (yellow and grey) represent the interaction between the nodes (minimum of 20% genes common between two connected) GO processes.

To further characterize the function of the wheat *dMTase* genes, we performed GO enrichment analysis using the ShinyGO tool (Ge et al., 2020). GO enrichment analysis showed that the *TadMTase* genes were involved in DNA demethylation, the BER pathway, DNA modification, developmental processes, and stress responses in GO biological processes (Figure 5A). The GO molecular process showed that *TadMTase* genes were involved in DNA demethylase activity, metal/Iron-sulphur cluster binding and catalytic activity (Figure 5B). This annotation indicates that the *TadMTase* genes may be involved in stress regulation in plants *via* DNA demethylation.

### Developmental Stage and Tissue-Specific Expression Patterns of TadMTases

**FIGURE 6:**
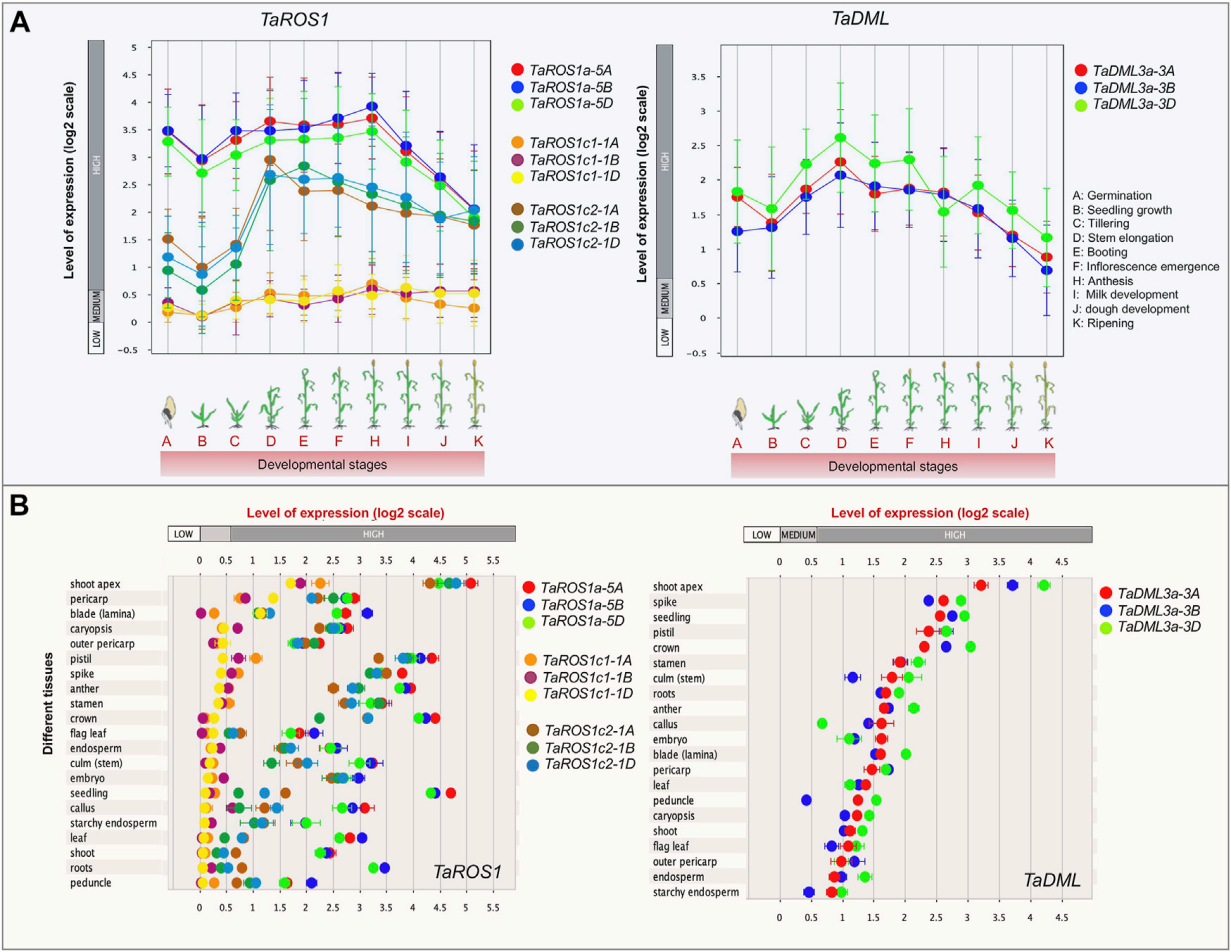
Expression profiles of wheat dMTase in the different developmental stages **(A)** and wheat tissues **(B)**. Showing the scatterplots for the 12 *TadMTase* genes, the left side in **(A)** and the topside in **(B)** represent the change of gene expression using the base 2 logarithm scale. Different dMTase genes are represented by distinct colors as indicated. Error bars represent standard errors. Data were analyzed with the Genevestigator tool.

To decipher the function of wheat *dMTase* genes, the expression pattern in ten developmental stages and 21 tissues was analyzed (Figure 6). The expression levels indicate that all the wheat *dMTase* genes were expressed in different developmental stages/tissues but varied significantly. During various developmental stages, the expression of dMTases gradually increased from the seedling to the booting stage and declined continuously till the ripening stages. However, *TaROS1a-5A/5B/5D* had the highest expression at anthesis stages, and *TaROS1c1/2 1A/1B/1D* and *TaDML3a 3A/3B/3D* had the highest expression at the stem elongation stage (Figure 6A). In the case of tissue-specific expression, *TaROS1a 5A/5B/5D*, *TaROS1c2 1A/1B/1D* and *TaDML3a 3A/3B/3D* exhibited relatively higher expression in all tissues. However, *TaROS1c1 1A/1B/1D* had tissue-specific expression and was expressed especially in the shoot apex, pericarp, and blade (lamina) tissues (Figure 6B). Furthermore, we also studied the expression of *TadMTase* genes in hormone-treated [abscisic acid (ABA) and gibberellic acid (GA3)] wheat tissues. *TaROS1a-5A/5B/5D* were downregulated (up to 1.96 fold) in root tissues at the seedling stage upon ABA treatment. Other genes do not show any significant change in their expression pattern (Supplementary Figure S3).

### Expression Patterns of TadMTases Under Heat Stress

**FIGURE 7:**
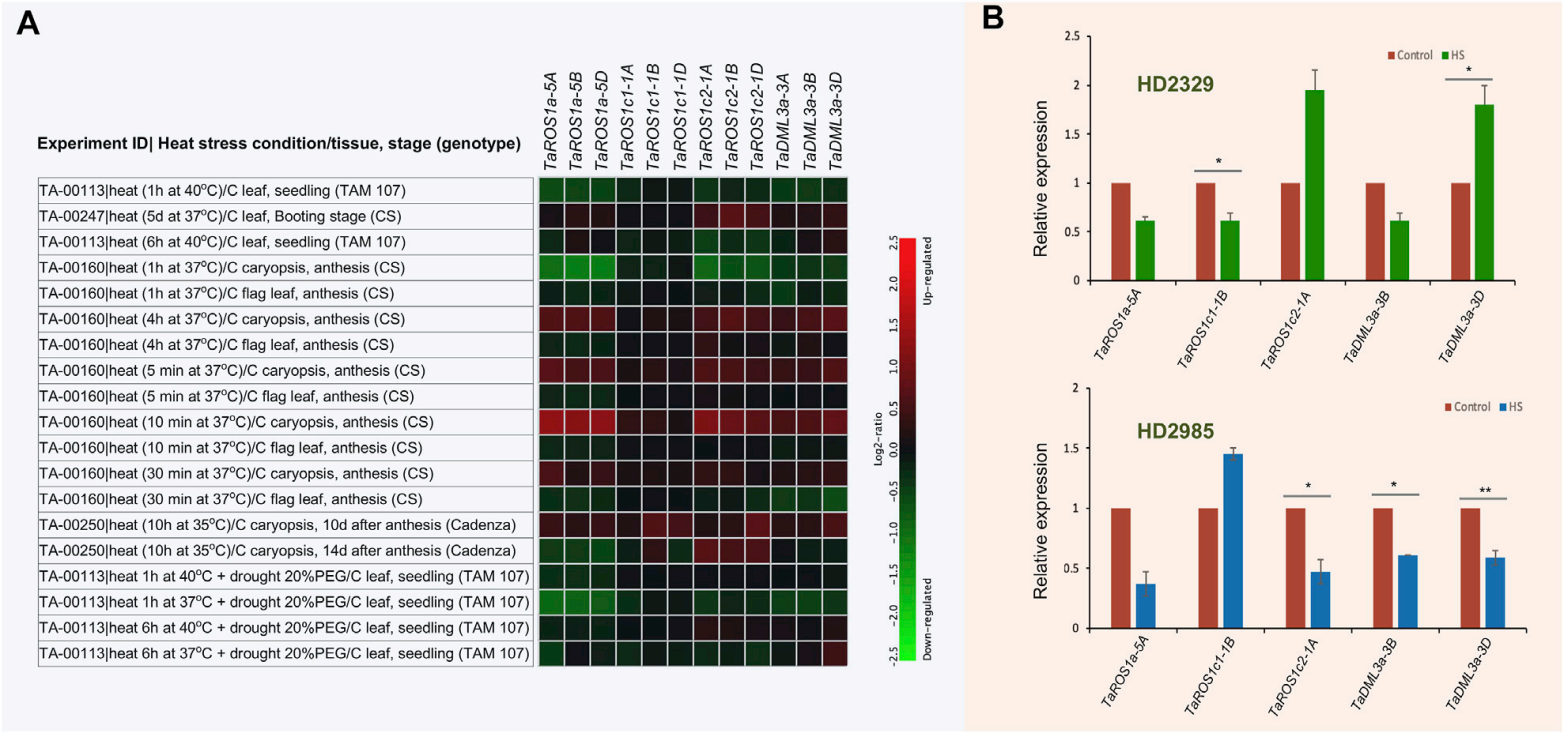
Expression profiles of wheat *dMTase* genes under heat stress (HS) conditions. **(A)** Heatmap showing the expression of 12 *TadMTase* genes in various tissues and developmental stages during heat stress conditions. The “heatmap” color represents relative expression values, calculated as log2 ratios between the signal intensities from HS treated genotypes vs. controls (C). The red color indicates up-regulation and the green color indicates downregulation. **(B)** The relative expression level of five *TadMTase* (*TaROS1a-5A, TaROS1c1-1B, TaROS1c2-1A, TaDML3a-3B, TaDML3a-3D*) genes was analyzed by quantitative RT-PCR in wheat seedling tissue under HS treatments (42°C for 2 h). Each bar value represent the means (±SE) derived from three biological. Asterisks on the bar showed significant differences between HS treated and control samples (*p < 0.05, **p < 0.01, Student’s *t*-test). CS, Chinese spring; PEG, poly (ethylene glycol).

Heat stress is one of the major stresses that severely affects wheat productivity. Therefore, there has been recent research to understand the mechanism by which plants respond to HS and identify the genes associated with HS tolerance (Samtani et al., 2022). To investigate the putative biological role of *TadMTase* under HS, the expression pattern of these genes under HS was determined using wheat expression data available in the Genevestigator tool and by performing the qRT-PCR experiments. The wheat RNA-seq data (available on the Genevestigator tool) of the four different experiments when were subjected to ambient (22°C) and heat stress (37°C–40°C) treatment for 5 min to 5 days (as shown in Figure 7A) were evaluated. Results showed that there were significant differential expression patterns and most of the *TadMTase* genes were suppressed (up to 2.24 folds) in response to HS as compared to controls (Figure 7A). For instance, in response to HS, *TaROS1a-5A, -5B,* and *-5D*, and *TaROS1c2-1A*, *-1B,* and *-1D* were downregulated in the seedling stage (leaf and caryopsis tissue; up to ∼2.3 fold) and *TaDML3a-3A*, *-3B,* and *-3D* were downregulated in the anthesis stage (flag leaf tissue; up to ∼2 fold).

Furthermore, we selected five genes (*TaROS1a-5A*, *TaROS1c1-1B*, *TaROS1c2-1A*, *TaDML3a-3B*, *TaDML3a-3D*) out of the 12 *TadMTase* genes for quantitative real-time PCR analysis. For this purpose, contrasting wheat genotypes (sensitive cv. HD2329 and tolerant cv. HD2985) were used. All the five *TadMTase* genes showed considerable differential expression after being stimulated by HS (Figure 7B). The expression levels of *TaROS1a-5A* and *TaDML3a-3B* were repressed (up to 2.7-fold) in both the genotypes. The expression levels of *TaROS1c2-1A* and *TaDML3a-3D* were repressed (up to 2.12-fold) in HS tolerant cv. HD2985 and upregulated in HS sensitive cv. HD2329. The expression level of *TaROS1c1-1B* was upregulated in HS tolerant cv. HD2985 and repressed in HS sensitive cv. HD2329 (Figure 7B). The above results indicate that active demethylation is possibly involved in wheat’s responses to thermal stress. Furthermore, the differentially regulated *TadMTase* might regulate thermotolerance in crops such as wheat.

## Discussion

DNA methylation is an important epigenetic mark and assists plants to adapt to different abiotic stress conditions (Lämke and Bäurle, 2017; Korotko et al., 2021). DNA methylation negatively controls the transcription of genes and the transposition of transposable elements (TEs) in plants (Matzke and Mosher, 2014). Furthermore, the DNA methylation state on specific sites depends on the following three activities: 1) establishment, 2) maintenance, and 3) active DNA demethylation. In plants, active DNA demethylation is performed by DNA demethylases genes (*ROS1, DML, and DME*) *via* the BER pathway (Zhu, 2009; Mok et al., 2010; Zhang et al., 2018). The model plant Arabidopsis encodes four different dMTases, namely *ROS1*, *DME*, *DML2*, and *DML3* (Zhang et al., 2018). The *dMTase* gene family has been detected and characterized in the following plant species, such as *Solanum lycopersicum* (Cao et al., 2014), *Arachis hypogaea* (Wang et al., 2016), *Fragaria vesca* (Gu et al., 2016), *Cynara cardunculus* var. *scolymus* (Gianoglio et al., 2017), *Pyrus bretschneideri* (Liu et al., 2018a)*, Ricinus communis* (Victoria et al., 2018), cotton (*Gossypium hirsutum, Gossypium arboretum, Gossypium raimondii,* and *Gossypium barbadebse*) (Yang et al., 2019) *Actinidia chinensis* (Zhang et al., 2020), *Camellia Sinensis* (Zhu et al., 2020), *Dendrobium officinale* (Yu et al., 2021). However, no comprehensive identification and characterization of these genes in wheat has been reported. Here, by utilizing the wheat reference genome sequence data (available at https://plants.ensembl.org/index.html), we have identified 12 *dMTases* (9 *ROS1* and 3 *DML*) genes in the wheat (Table 1). The phylogenetic relationships, gene structures, CREs, conserved domains and motifs, SSR motifs, and expression profiling in various development stages/tissues and during HS for TadMTases were also analyzed.

As per our findings, wheat has a higher number of dMTases than some other plant species. In different plant species, it varies from 2 (*Vitis vinifera*) to 10 (*Gossypium hirsutum*). This disparity might be attributable to wheat’s allohexaploid nature. Based on the corresponding genes identified for Arabidopsis and rice, as well as phylogenetic analyses, these dMTase genes were divided into two groups (ROS1 and DML). The DME group was not found in wheat and other monocots (Figure 1B), and earlier studies have also discovered that DME was only detected in eudicots and absent in monocots (Choi et al., 2002; Zemach et al., 2010; Yu et al., 2021). The role of DME genes in monocots must be supplemented by ROS1 or DML orthologs. Furthermore, the absence of DME genes in wheat and in other monocots suggests that DMEs are a recently developed type of DNA demethylase gene in dicots.

The phylogenetic analysis showed that 59 dMTase proteins belonging to 10 species were grouped into three distinct subfamilies, that is, ROS1, DME, and DML (Figure 1A). Within each subfamily, wheat dMTases showed a closer phylogenetic relatedness to a tier of wild relative species. For example, *TaROS1a-5a* and *TtROS1-5A* (*Triticum turgidum*) and *TaDML3a-3A* and *TuDML3a* (*Triticum Urartu*) had a close evolutionary relationship (Figure 1A). We also observed that in the ROS1 and DML subfamilies, monocotyledons and dicotyledons formed distinct subgroups. The DME subfamily was only found in dicots and absent in monocots, which suggests the loss of DME genes during monocot evolution (Figure 1B). Furthermore, wheat dMTases had similar exon-intron, motif, and domain organization throughout each subfamily (Figures 2, 3). Exon-intron, motif, and domain organizations were discovered to be varied amongst the sub-families. Similar observations for dMTase genes were found in several other plant species (Wang et al., 2016; Zhang et al., 2020; Zhu et al., 2020; Yu et al., 2021).

NLS analysis has shown that most of the TadMTases have NLS domains (Supplementary Table S4), which are required for the precise targeting of these proteins to the nucleus. The sub-cellular localization studies also found that wheat dMTases were positioned in the nucleus (Table 1). The dMTases in the following plant species, that is, Arabidopsis (Gong et al., 2002), tomato (Cao et al., 2014), peanut (Wang et al., 2016), globe artichoke (Gianoglio et al., 2017), castor bean (Victoria et al., 2018), eggplant (Moglia et al., 2019), tea plant (Zhu et al., 2020), and orchid (Yu et al., 2021) were also shown to be localized in the nucleus. Considering the importance of subcellular location in defining a protein’s function, these results indicate that the function of *dMTase* genes in plants may be conserved.

Gene-based SSR markers are a highly valuable resource and could be used for functional diversity analysis, comparative mapping, evolutionary analysis, and molecular plant breeding (Varshney et al., 2005). Gene-based SSR markers were developed in several crop plants like rice (Molla et al., 2015), barley (Zhang et al., 2014), wheat (Kumar et al., 2018; Singh et al., 2018; Mehta et al., 2021), millet (Desai et al., 2021) and many others. For wheat *dMTase* genes, we also identified 17 SSR motifs and designed primer pairs (Supplementary Table S3). These SSR markers may be examined in different wheat genotypes for polymorphism, and the polymorphic SSRs may be utilized for molecular breeding (i.e., marker-assisted selection, marker-assisted recurrent selection) in wheat breeding program for the improvement of different agronomic traits and for heat stress tolerance.

*Cis*-regulatory elements (CREs) are known to play a role in the modulation of a variety of biological processes, including abiotic stresses (Azodi et al., 2020). We also found abiotic stress-responsive, hormonal regulation related, light-responsive, and plant development-related CREs in the promoter of *TadMTase* genes in this investigation (Figure 4). Similar types of CREs have been reported in the promoter regions of dMTase genes in other plants. For instance, CAAT, G-box, TCA, ABRE elements, etc., were reported in the promoter of dMTase genes belonging to *Arachis hypogaea* (Wang et al., 2016), *Actinidia chinensis* (Zhang et al., 2020), *Camellia Sinensis* (Zhu et al., 2020), and *Dendrobium officinale* (Yu et al., 2021). According to these findings, wheat dMTase genes may have a role in the development and stress response regulation in plants. Furthermore, in wheat, we also observed that the number of CREs and their distribution patterns differ between the promoters of three wheat homoeologous genes (Figure 4). On the other hand, their encoding proteins had similar domains and amino acid sequences. This highlighted the homologous genes’ distinct regulatory activities during wheat polyploidization.

DNA methylation regulates an array of biological processes that control plant growth and development as well as biotic and abiotic stress responses (Bender, 2004; Zhang et al., 2018). DNA methylation maintenance also requires the active participation of dMTases, which remove C methylation (Mok et al., 2010). Furthermore, several studies have revealed that dMTases are intricate in the regulation of growth and development, either directly or indirectly. For instance, *DML3* was reported to be expressed during leaf senescence in Arabidopsis and regulates the leaf senescence process (Yuan et al., 2020). The *OsROS1a* knock-in null mutation affects the endosperm development and causes the formation of irregular embryos (Ono et al., 2012). DNA demethylation mediated by *OsROS1a* can alter the number of aleurone layers (Liu et al., 2018b). Herein, in wheat, we also found that dMTase genes exhibited significantly differential expression in different tissues and developmental stages (Figure 6). For instance, TaROS1a had the highest expression at the anthesis stages, and TaROS1c and *TaDML3a* had the highest expression at the stem elongation stage (Figure 6A). In tissue-specific expression analysis, it was observed that *TaROS1a* and *TaDML3a* had relatively higher expression in all tissues. Similar results were also noticed in other plant species. For instance, ROS2 like in peanut (Wang et al., 2016), *SmelDemethylase_5* in eggplant (Moglia et al., 2019) and *DoDML3* in *Dendrobium officinale* (Yu et al., 2021), had relatively higher expression in all the tissues. However, *TaROS1c1* had tissue-specific expression and was expressed especially in the shoot apex, pericarp, and blade (lamina) tissues (Figure 6B).

It was reported that the active demethylation phenomenon was involved in HS regulation in plants (Naydenov et al., 2015; Korotko et al., 2021; Malabarba et al., 2021). In previous studies on strawberry (Gu et al., 2016) and cotton (Yang et al., 2019), demethylase gene expression was found to be modulated in response to HS, implying that it may have a role in the plant’s response to HS. We analyzed the expression profiles of wheat *dMTase* genes under HS conditions. Our results revealed that there are significant differential expression patterns of *TadMTase* genes and most of them were suppressed in response to HS as compared to controls. These findings were then confirmed by using the qRT-PCR method for the following five genes (*TaROS1a-5A, TaROS1c1-1B, TaROS1c2-1A, TaDML3a-3B, TaDML3a-3D*). During the HS condition, all the five *TadMTase* genes showed considerable differential expression in response to HS (Figure 7B). For example, *TaROS1a* and *TaDML3a* expression levels were suppressed in both genotypes. The expression levels of *TaROS1c2 and TaDML3a* were reduced in HS tolerant cv. HD2985 and induced in HS sensitive cv. HD2329 (Figure 7B). Interestingly, these genes also contain stress-responsive CREs in their promoter region, that is, ARE, CAAT-box, CCAAT-box, GC-motif, LTR, and MBS, which are associated with various abiotic stresses, including HS (Figure 4). These findings suggest that *TadMTase* genes may have a variety of roles in plants, including plant growth and development as well as HS control. However, further functional research is required to corroborate the findings of this study.

## Conclusion

A comprehensive and systematic analysis was conducted, and 12 dMTase genes were identified in the wheat genome. Each *TadMTase* member is comprised of three conserved domains (Domain A, HhH-GPD glycosylase domain, and RRM_DME domain). The study also analyzed chromosomal distributions, evolutionary relationships, gene and protein structures, NLS and subcellular localization, CREs, and SRR motifs. Differential expression profiles of TadMTases in different tissues and developmental stages suggest that they are essential in plant growth and development. Furthermore, differential gene expression in response to HS conditions revealed a role for these genes in the wheat’s stress response. Future studies on the role of dMTases in plant development and abiotic stresses *via* DNA methylation might add to our understanding and help us improve wheat.

## Data Availability

The original contributions presented in the study are included in the article/Supplementary Material; further inquiries can be directed to the corresponding author.
