# Supplementary material for: Identification and Characterization of DNA Demethylase Genes and Their Association With Thermal Stress in Wheat (Triticum aestivum L.)
Source: Front Genet. 2022 Jul 22;13:894020. doi: 10.3389/fgene.2022.894020 (PMC9355123; doi:10.3389/fgene.2022.894020)
Supplement: Supplementary file 1 [file DataSheet1.PDF]

Supplementary Material

Supplementary Figure S1. Locations of wheat dMTase genes on different chromosomes. Dotted lines indicate a homeologous relationship. The scale is in megabases (Mb).

Supplementary Figure S2. Multiple sequence alignment analysis of the 12 wheat dMTase protein sequences and an Arabidopsis dMTase protein sequence. The key conserved domains are highlighted by double lines (Domain A by green colour, HhH-GPD glycosylase domain by red colour and RRM_DME domain by brown colour) and the domain names are shown at the top of the sequence. Names of genes are shown on the left.

Supplementary Figure S3. Heatmap showing expression of 12 TadMTase genes in various tissues and developmental stages during hormonal stress (ABA and GA3). The "heatmap" colour represents relative expression values, calculated as log2 ratios between the signal intensities from hormonal treated genotypes vs. controls (C). The red colour indicates up-regulation and the green colour indicates down-regulation. ABA, abscisic acid; gibberellic acid, GA3.

Supplementary Table S1. Primer pairs used for qRT-PCR analysis.

Supplementary Table S2. Sequence homology (BLASTP) of dMTase genes between wheat, Arabidopsis and rice

Supplementary Table S3. List of SSR markers along with the following details, type of repeat motif, repeat position, primer sequences, Tm (^{o}C) and product size(bp).

Supplementary Table S4. Details of Monopartite and bipartite nuclear localization signals (NLS) predicted in TadMTases (cut-off score = 5).

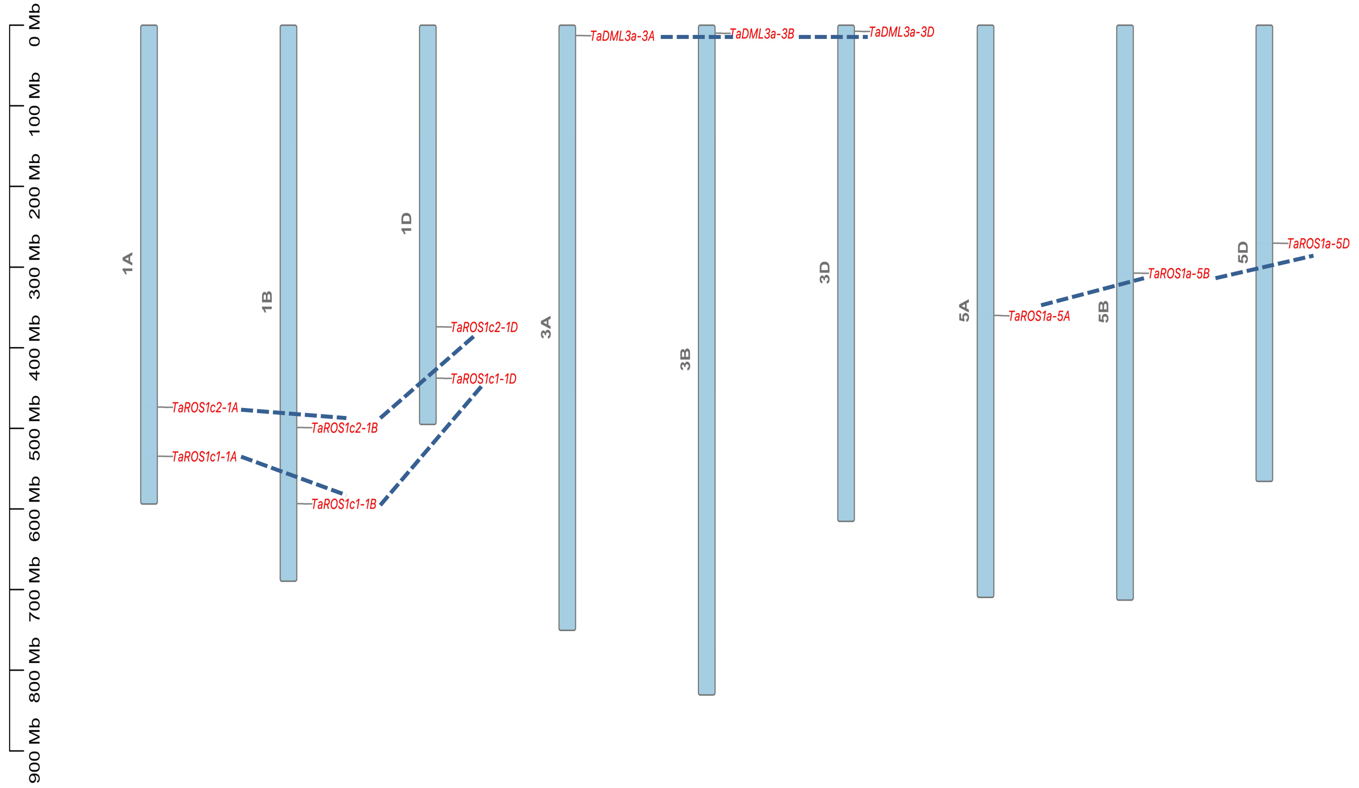

Supplementary Figure S1. Locations of wheat dMTase genes on different chromosomes. Dotted lines indicate a homeologous relationship. The scale is in mega bases (Mb).

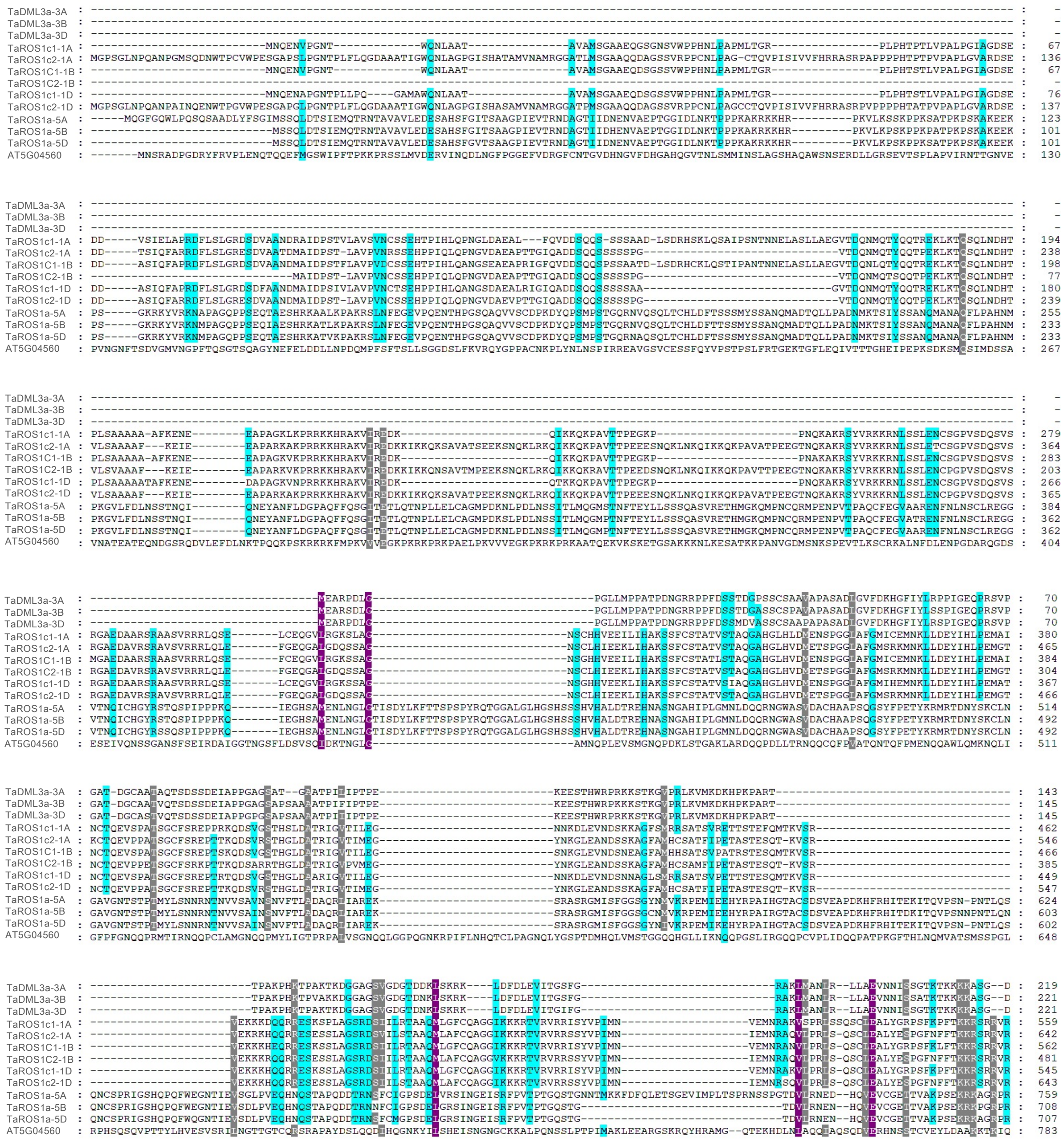
TaDML3a-3A
TaDML3a-3B
TaDML3a-3D
TaROS1c1-1A
TaROS1c2-1A
TaROS1C1-1B
TaROS1C2-1B
TaROS1c1-1D
TaROS1c2-1D
TaROS1a-5A
TaROS1a-5B
TaROS1a-5D
AT5G04560

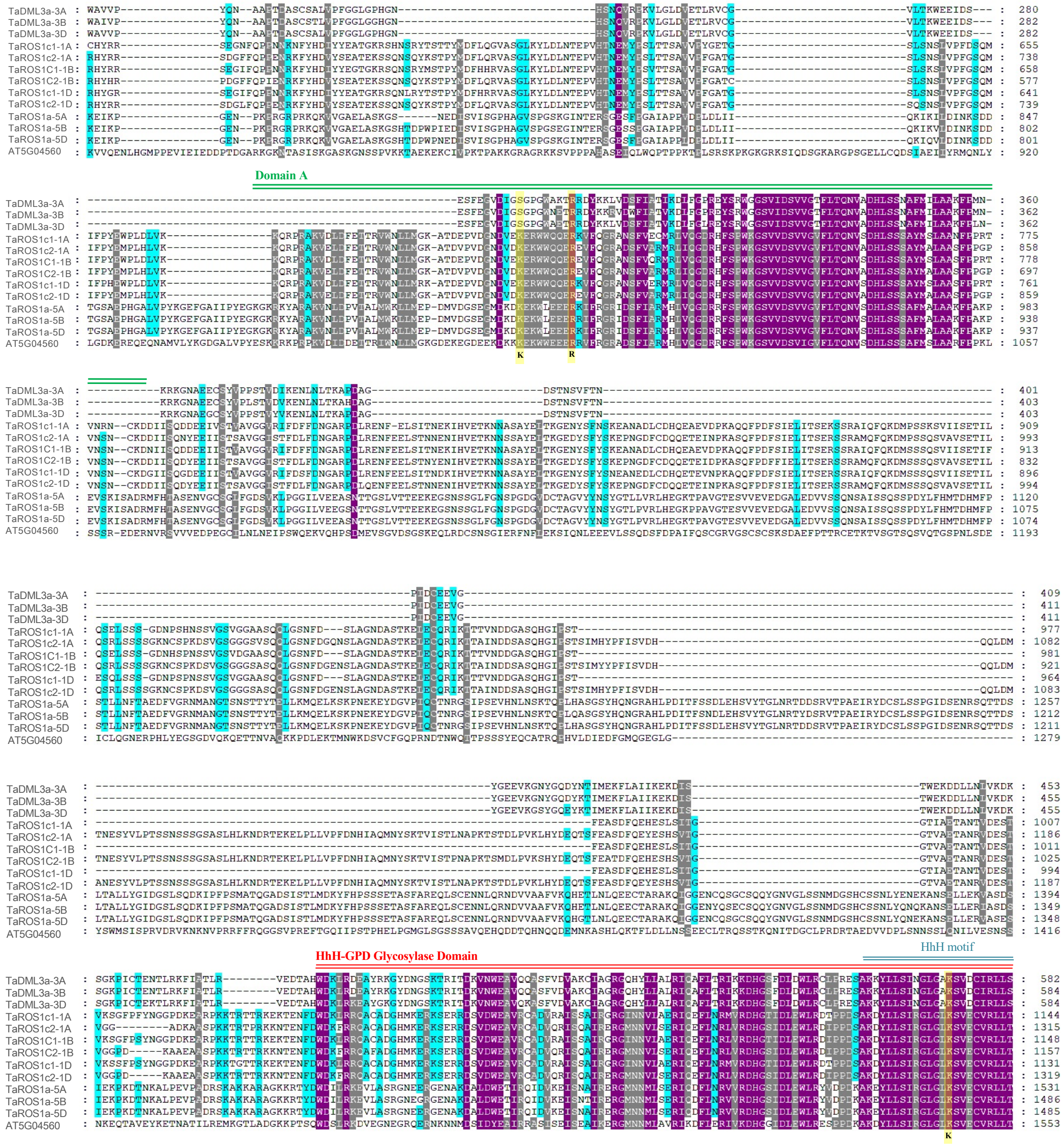
Domain A
HhH-GPD Glycosylase Domain
HhH motif
TaDML3a-3A
TaDML3a-3B
TaDML3a-3D
TaROS1c1-1A
TaROS1c2-1A
TaROS1C1-1B
TaROS1C2-1B
TaROS1c1-1D
TaROS1c2-1D
TaROS1a-5A
TaROS1a-5B
TaROS1a-5D
AT5G04560
K
R

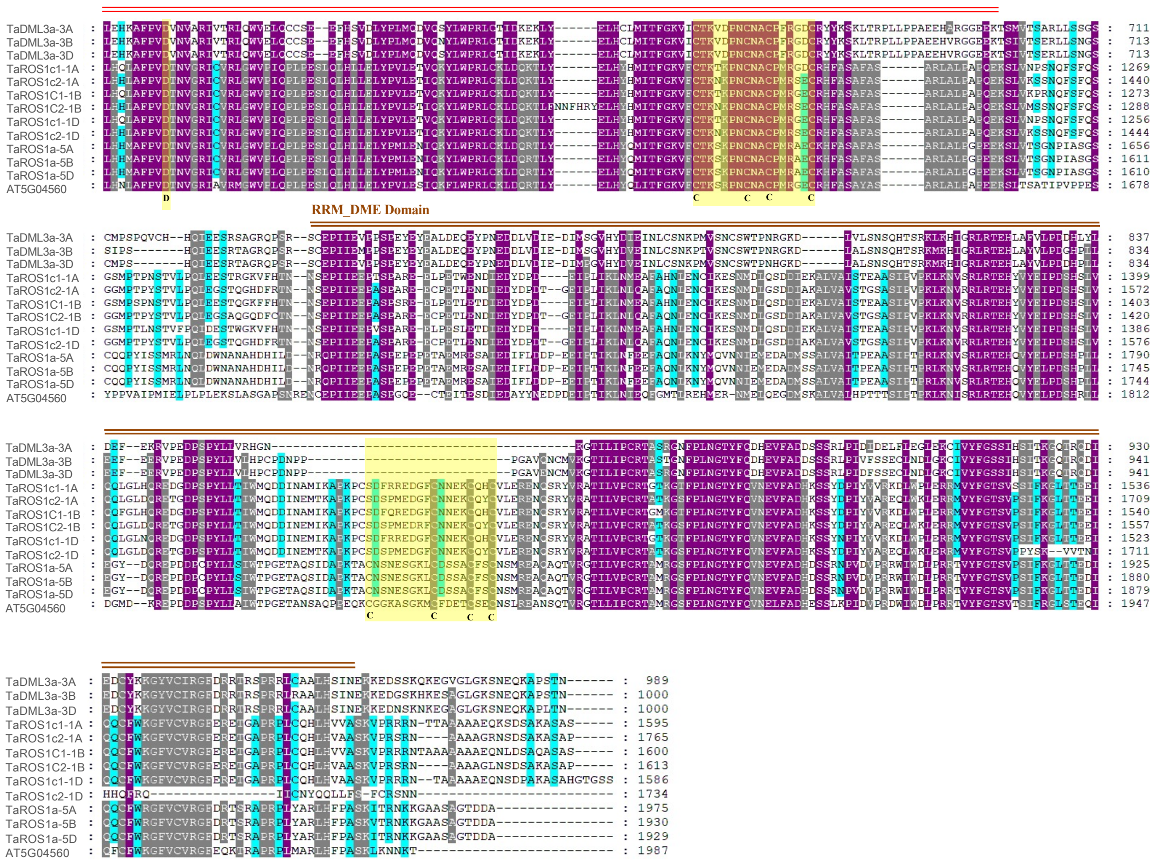

Supplementary Figure S2. Multiple sequence alignment analysis of the 12 wheat dMTase protein sequences and an Arabidopsis dMTase protein sequence. The key conserved domains are highlighted by double lines (Domain A by green colour, HhH-GPD glycosylase domain by red colour and RRM_DME domain by brown colour) and the domain names are shown at the top of the sequence. Names of genes are shown on the left.

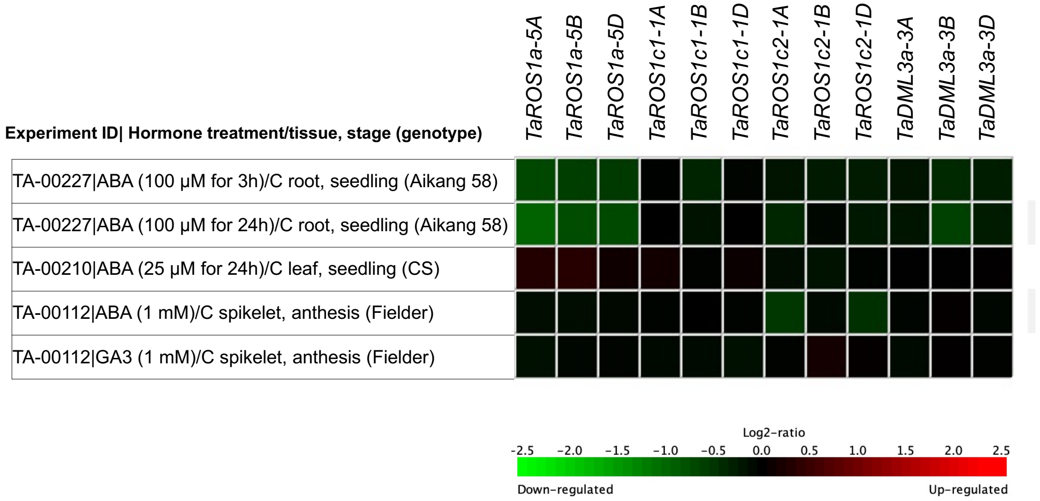

Supplementary Figure S3. Heatmap showing expression of 12 TadMTase genes in various tissues and developmental stages during hormonal stress (ABA and GA3). The "heatmap" colour represents relative expression values, calculated as log2 ratios between the signal intensities from hormonal treated genotypes vs. controls (C). The red colour indicates up-regulation and the green colour indicates down-regulation. ABA, abscisic acid; gibberellic acid, GA3.

Supplementary Table S1. Primer pairs used for qRT-PCR analysis.

Gene	Forward sequence (5'-3')	Reverse sequence (5'-3')
TaROS1a-5A	ATTGCTCGCATGCATCTAGTTC	AAACACCCACTACTGAATCCACAA
TaROS1c1-1B	TTGCATCAGGGCTGAAATATCTT	GGATGTGGTCACAGATGGATACA
TaROS1c2-1A	GGCCATCCGGGAACGA	CCATGATCTCTAACCAAGCGATT
TaDML3a-3B	TGAAGAGCGAGTTCCAGAAGATC	CAGCACCAGGAGGAGGATTG
TaDML3a-3D	TTTGAAGAGCGAGTTCCAGAAGA	GCACCAGGAGGAGGATTGTC
Wheat GADPH	GGTGCCAAGAAGGTCATCAT	TGGTCATCAAACCCTCAACA

Supplementary Table S2. Sequence homology (BLASTP) of dMTase genes between wheat, Arabidopsis and rice

Wheat		Arabidopsis		E-Value	Identities (%)	Rice		E-Value	Identities (%)
Gene Name	Gene ID	Gene Name	Gene ID			Gene Name	Gene ID		
TaROS1a-5A	TraesCS5A02G169000	*AtROS1*	AT2G36490	0	65.2	*OsROS1a*	Os01t0218032	3.90E-164	87.5
TaROS1a-5B	TraesCS5B02G165800	*AtROS1*	AT2G36490	0	62.9	*OsROS1a*	Os01t0218032	3.90E-164	87.5
TaROS1a-5D	TraesCS5D02G173300	*AtROS1*	AT2G36490	0	61.0	*OsROS1a*	Os01t0218032	5.30E-164	87.1
TaROS1c1-1A	TraesCS1A02G349600	-	-	-	-	*OsROS1c*	Os05t0445900	2.30E-137	82.7
TaROS1c1-1B	TraesCS1B02G364100	-	-	-	-	*OsROS1c*	Os05t0445900	2.30E-137	82.5
TaROS1c1-1D	TraesCS1D02G352500	-	-	-	-	*OsROS1c*	Os05t0445900	3.60E-135	81.4
TaROS1c2-1A	TraesCS1A02G278000	-	-	-	-	*OsROS1c*	Os05t0445900	8.60E-144	85.7
TaROS1c2-1B	TraesCS1B02G286900	-	-	-	-	*OsROS1c*	Os05t0445900	1.20E-143	83.8
TaROS1c2-1D	TraesCS1D02G277100	-	-	-	-	*OsROS1c*	Os05t0445900	1.00E-145	84.2
TaDML3a-3A	TraesCS3A02G022500	*AtDML3*	AT4G34060	3.70E-27	52.0	*OsDML3a*	Os02t0496500	1.30E-56	59.6
TaDML3a-3B	TraesCS3B02G023200	*AtDML3*	AT4G34060	9.50E-27	52.0	*OsDML3a*	Os02t0496500	4.50E-59	62.1
TaDML3a-3D	TraesCS3D02G024100	*AtDML3*	AT4G34060	6.90E-27	52.0	*OsDML3a*	Os02t0496500	1.10E-54	59.6

Supplementary Table S3. List of SSR markers along with the following details, type of repeat motif, repeat position, primer sequences, Tm (°C) and product size(bp).

Gene Name	(Motif) repeats	Position	Primer details			
			Forward Primer (5'-3')	Reverse Primer (5'-3')	Tm (°C)	Product size (bp)
TaROS1a-5A	(AAAT)3	12464-75	AAGGAAAACTGTGGGAATTGG	GCCTTGCATAGAGTGGTCTTG	60.05	288
TaROS1a-5B	(ATTT)3	8045-56	CCGTTCACTCGGGAAGTTT	TCCCAGCCTCACACAAATC	59.55	361
	(TA)19	13536-73	ACAGCTCCCAAGAGGATAAACA	CTACCCCATTATAGCTCCCTCC	60.15	198
TaROS1a-5D	NA	-	-	-	-	-
TaROS1c1-1A	(CAG)4	1051-62	GCCAAATGGTTTAGATGCAGA	GGCTCTATGTTTCTTCCTCCG	60.15	322
	(CAG)6	11575-92	TTCCCTGACAAAGAGTGTGATG	TCTAAAATCAGCCCCAAACAAC	60.24	342
TaROS1c1-1B	(GAAA)3	4392-404	ATCACTGGTGGAACGGTAGC	GATTCGCTCTGCCAAAACAT	60.11	351
	(AAAT)3	7305-17	TTGGATATTCTGGCTCAATGC	CAAACACAGGCCAACACAAAT	60.44	329
	(CAG)8	12326-49	TTGCTTCCAGACTTATTTGGGT	AACTAACTAATACGGCCCATGC	59.72	321
TaROS1c1-1D	(CAG)6	110111-28	ACAAAGAGTGTGACGCGATG	CTGAGAACGTGAAAACGCTG	59.76	390
TaROS1c2-1A	(TCA)4	1163-74	TCTGAACATCCACCAATACAGC	CAGTGAGGCCAATTCATCAGTA	60.06	174
TaROS1c2-1B	(TCA)4	1113-24	ATGCAGAAGCACCCACAAC	AGGAAGCAGTGAGGCCAAT	59.75	140
	(AATAA)3	8032-46	AACCATGTTCTGACTCTCCGAT	ATCATGCAACAAACACAGGCTA	60.25	386
TaROS1c2-1D	(TCA)4	1112-23	TCTTCCGAGGTTGTATGTTGG	CAGTGAGGCCAATTCATCAGT	60.05	393
TaDML3a-3A	(CCCGC)3	106-20	TATCCCATTGTCCCTGCTG	ATCCGTGGAGGAATCGAAC	59.60	282
	(AG)8	5857-73	TCCTTATCTCCTGGTTCGTCAT	AGTTGCATACCTCAATGCTCCT	60.06	217
	(ATT)5	6298-311	CGTGCATCAACCATCTCATACT	GTTGTAAGAAGTGACCGTGCAG	59.92	241
TaDML3a-3B	(GCC)4	95-107	ATTGTCACTGCTGCTGCCT	CAGATTCGATCAAGAAGAAGGG	59.89	147
TaDML3a-3D	NA	-	-	-	-	-

Supplementary Table S4. Details of Monopartite and bipartite nuclear localization signals (NLS) predicted in TadMTases (cut-off score = 5).

Protein name	Position (aa)	NLS sequence	Score	Monopartite/bipartite
TaROS1a-5A	90	PPKAKRKKHRP	9.0	monopartite
	498	PETYKRMRTDNY	7.0	monopartite
	749	PSEKRKAGRP	5.0	monopartite
TaROS1a-5B	68	PPKAKRKKHRP	9.0	monopartite
	476	PETYKRMRTDNY	7.0	monopartite
	106	RKYVRKNMPAGQPPSEQIAESHRKATLKPAKRS	7.0	bipartite
	128	RKATLKPAKRSLNFEGEVPQENTHPGSQAQVVSCDP	5.2	bipartite
TaROS1a-5D	68	PPKAKRKKHRP	9.0	monopartite
	102	PSGKRKYVRK	5.0	monopartite
	476	PETYKRMRTDNY	7.0	monopartite
	697	PSEKRKAGRP	5.0	monopartite
	128	RKATVKPAKRSLNFEGEVPQENTHPGSQAQVVSCDP	5.2	bipartite
TaROS1c1-1A	215	LKPRRKKHRAK	7.5	monopartite
	550	FTKKRSRRVRCHYRRSEGNFQPPNNKNFYHD	6.0	bipartite
	1029	RTTRKEKTENFDWDKLRRQACADGHMKERKSE	5.5	bipartite
TaROS1c1-1B	219	VKPRRKKHRAK	7.5	monopartite
	1033	RTTRKEKTENFDWDKLRRQACADGHMKERKSE	5.5	bipartite
TaROS1c1-1D	202	VNPRRKKHRAK	7.5	monopartite
	536	FTKKRSRRVRRHYGRSEGIFQPPNNRKFYHD	5.0	bipartite
	1017	TTRKEKTENFDWDKLRRQACADGHMKERKSE	5.3	bipartite
TaROS1c2-1A	259	PRRKKHRAK	6.5	monopartite
	1200	RTPRKKNTENFDWDKFRRQACADGHMKERKSE	5.4	bipartite
TaROS1c2-1B	96	VKPRRKKHRAK	7.5	monopartite
	1042	RTTRKKNTENFDWDKFRRQAFADGHMKERKSE	5.4	bipartite
TaROS1c2-1D	260	PRRKKHRAK	6.5	monopartite
	1204	RTTRKKNTENFDWDKFRRQACADGHMKERKSE	5.2	bipartite
TaDML3a-3A	169	DKLSKRKLDFD	5.0	monopartite
	358	PMNKRKGNAE	6.0	monopartite
	118	RPRKKSTKGVPRLKVMKDKHPKPARTTPAKPHKTP	5.5	bipartite
TaDML3a-3B	172	KLSKRKLDFD	6.0	monopartite
	360	PMNKRKGNAE	6.0	monopartite
	144	RTTPAKPHKTPVAKKDGGAGSVGDGTDNKLSKRK	6.7	bipartite
TaDML3a-3D	172	KLSKRKLDFD	6.0	monopartite
	360	PLNKRKGNAE		monopartite
	144	RTTPAKPHKTPAKTKDGGAGSVGDGTDNKLSKRK	7.5	bipartite
